# Comparison of the effects of 0.05% topical cyclosporine A versus 0.1% topical cyclosporine A on recurrence and clinical parameters following pterygium surgery

**DOI:** 10.55730/1300-0144.5836

**Published:** 2024-02-12

**Authors:** Emin Serbülent GÜÇLÜ, Tamer METİN, Ömer ÖZER, Fatma Merve BEKTAŞ

**Affiliations:** 1 Department of Ophthalmology, Mersin City Training and Research Hospital, Mersin, Turkiye; 2 Department of Ophthalmology, Faculty of Medicine, Niğde Ömer Halisdemir University, Niğde, Turkiye

**Keywords:** Cyclosporine A 0.05%, cyclosporine A 0.1%, inflammation, pterygium, recurrence

## Abstract

**Background/aim:**

To compare the efficacy of topical 0.05% cyclosporine A (CsA) and 0.1% topical cyclosporine A (CsA) over a 6-month period following pterygium surgery, specifically evaluating their effects on postoperative recurrence and clinical parameters.

**Material and methods:**

This clinical study enrolled 245 patients with pterygium who underwent surgery using the conjunctival autograft technique with mitomycin C (MMC) were enrolled. Participants were divided into three groups: Group 1 (0.05% CsA) (n = 80), Group 2 (0.1% CsA) (n = 80), and a control group (n = 85). They were examined at postoperative first day, first week, first month and sixth month. The examination included best corrected visual acuity (BCVA), intraocular pressure (IOP), presence of inflammation, and ptergium recurrence, all of which were compared across the groups.

**Results:**

The mean age of the patients was 63.22 ± 9.39 years, with 53.3% male and 46.7% female. The three groups were similar in terms of demographic characteristics and pterygium size. Inflammation in surgical area significantly regressed in all groups at 6 months postoperatively (p < 0.05). Inflammation in the first and sixth months was not different between the groups (p = 0.118, p = 0.580, and p = 0.435, respectively). The recurrence rate was not different between groups (p = 0.890). There was no statistically significant difference between groups regarding IOP (p = 0.818). A significant increase in BCVA after surgery was observed in three groups compared to preoperative levels (p < 0.05).

**Conclusion:**

This study showed that there was no difference between the efficacy of 6 month topical 0.05% CsA and 0.1% CsA application after pterygium surgery with the conjunctival autograft technique with MMC on postoperative outcomes. Including postoperative recurrence, IOP changes, BCVA changes and surgical area inflammation.

## 1. Introduction

Pterygium is a degenerative and proliferative ocular surface disease characterized by fibrovascular extension of the conjunctiva onto the cornea. Although the exact etiology is unknown, it is strongly associated with exposure to ultraviolet light. It occurs all over the world, but it is more common in dusty, sunny, hot climates and subtropical regions. Its frequency increases between 37 degrees south and north latitudes, also called the pterygium belt [1]. Ultraviolet light, believed to cause pterygium, can induce chronic inflammatory cells in the conjunctiva and damage limbal stem cells. Therefore, chronic inflammation contributes to the formation of pterygium [2–4]. Pterygium can cause nonaesthetic appearance, redness, foreign body sensation, itching, and dryness. Sometimes it can spread to the entire corneal surface and close the visual axis, causing vision loss. Involvement of the visual axis, high astigmatism, diplopia due to ocular motility limitation, and cosmetic causes are indications for treatment in pterygium. The treatment of pterygium is surgery. The aim of surgery is to provide ocular surface reconstruction and to prevent recurrence as much as possible [5].

The most important risk of pterygium surgery is the high risk of recurrence, which may vary according to the patients and surgical methods. Surgical methods include bare sclera rotational flap technique, conjunctival autograft technique and amnion membrane transplantation techniques [6–8]. Accordingly, intraoperative and postoperative use of adjuvant agents such as mitomycin C and 5-fluorouracil has been suggested; however, their effectiveness in reducing recurrence remains uncertain, and their side effects may be significant [9] Due to the role of inflammation in pterygium formation, attention has also been drawn to antiinflammatory agents [10].

Cyclosporine A (CsA), one of these antiinflammatory agents, is a calcineurin inhibitor. It is an antiinflammatory agent that suppresses T-helper cells, controls interleukin synthesis, and inhibits vascular endothelial growth factor [11]. CsA can also suppress the transition from fibroblast to myofibroblast through inhibition of myofibroblast markers induced by transformed growth factor-beta 2 [12].

In a metaanalysis comparing the recurrence rate, the use of conjunctival autograft combined with 0.05% CsA eye drops was associated with a lower recurrence rate [13]. However, some studies suggested that CsA did not have a significant effect on the recurrence rate [14–16].

We aimed to compare the efficacy of topical application of 0.05% CsA and 1% CsA over 6 months following pterygium surgery, focusing on postoperative recurrence and clinical parameters. To achieve this, we selected pterygium patients treated with MMC and the conjunctival autograft technique. The effectiveness of the postoperative treatments was then compared between the groups.

## 2. Materials and methods

Patients who were diagnosed with primary pterygium and had surgery indication in Mersin City Training and Research Hospital from January 2020 to January 2022 were enrolled in this clinical study. The study protocol was approved by Toros University Clinical Resarch Ethics Committee under decision number 185, dated November 28, 2022. Patients signed written informed consent. The Declaration of Helsinki was followed in this study.

Patients with primary pterygium of 3 mm or above on the horizontal axis were included in this study. Patients with recurrent pterygium, pseudoptergium, complicated pterygium surgery, keratitis, conjunctivitis and severe dry eye disease, ocular surgery within the last six months, diabetes mellitus and cardiac disease, history of hypersensitivity to CsA, as well as pregnant and breastfeeding women, were excluded. The patients were divided into three groups: Group 1 (0.05% CsA), Group 2 (0.1% CsA), and the control group (CsA not used).

All patients underwent a complete ophthalmological examination with a slit lamp biomicroscopy. Both preoperative and postoperative examinations were performed by the same surgeon. Demographic and preoperative data of the participants, including age and sex, were recorded. Preoperative measurements of best corrected visual acuity (BCVA) and intraocular pressure (IOP) were also taken. Slit lamp examinations were used to measure and grade of the pterygium size. The pterygium size was defined as the distance from the corneal limbus to the head of the pterygium. The pterygium was graded according to the Tan classification [17]. Tan staging is a classification based on the visibility of episcleral vessels under the pterygium tissue. According to this classification, stage 1 is atrophic, with clearly visible episcleral vessels. In stage 2, the episcleral vessels are moderately visible and partially distinguishable. Stage 3 is characterized by an opaque appearance, where episcleral vessels cannot be distinguished.

All patients underwent pterygium surgery using the conjunctival autograft technique with MMC. All surgeries were performed by the same surgeon. The surgical procedure was performed under local anesthesia. Initially, 0.5% topical proparacaine (Alcaine, Alcon, USA) was instilled. A speculum was placed, and 0.5 mL of local anesthetic lidocaine hydrochloride 20 mg/mL was injected into the pterygium body with a 27 gauge needle. The pterygium body was then grasped with the tenon capsule and cut with the help of scissors. Hemostasis was achieved by thermal cautery. A sterile cotton-tipped applicator moistened with 0.02% MMC (Kyowa, Seoul, Korea) was applied to the scleral bed for 90 s. Abundant irrigation was performed with 200 mL of normal saline solution. A conjunctival autograft was prepared from the superotemporal bulbar conjunctiva under the upper eyelid. The graft was attached with tissue adhesive (Tisseel, Baxter, USA) and fixed to the conjunctiva and sclera. In some patients with poor compliance, the graft was supported with 8/0 polyglactin 910 absorbable and synthetic suture. Suture removal was performed one month later.

All groups received moxifloxacin 0.5% (Moxai, Abdi Ibrahim, Türkiye) four times a day for 4 weeks, dexamethasone 0.1% (Maxidex, Novartis, Switzerland) four times a day for 1 month, and preservative-free artificial tear drops (Eyestil, SIFI, Italy) four times a day for 1 month. In addition to these medications, Group 1 received 0.05% CsA (Depores, Deva, Türkiye) twice a day for 6 months, while Group 2 received 0.1% CsA (Depores X, Deva, Türkiye) twice a day for 6 months. Patients were examined on the first postoperative day, at one week, 1 month, and 6 months. Slit lamp examinations were performed at each visit, and signs on inflammation in the surgical area, BCVA, IOP, and postoperative recurrence were recorded.

Postoperative inflammation was defined as hyperemia of the surgical area. The degrees of inflammation and recurrences were graded using the classification proposed by Prabhasawat, as follows: G0 for absence of inflammation, G1 for mild inflammation, G2 for moderate inflammation, and G3 for severe inflammation. Grades 2 and 3 are considered recurrence [18].

All statistical analyses were performed using the statistical software IBM SPSS Statistics for Windows version 21.0 (IBM Corp. Armonk, NY, USA). The normal distribution was tested with the Kolmogorov–Smirnov test, and according to its result, the mean ± standard deviation or median range was used for the definition of variables with and without normal distribution, respectively. Frequency (percentage) was used for categorical variables. Comparisons between the three groups were made using Student’s t-test and Mann–Whitney U for normally distributed numerical variables. Chi-square or Fisher’s precision test was used according to the sample size in the comparison of categorical variables. Wilcoxon signed-rank test or paired-sample t-test was used to compare the differences in each study group for normally distributed and nonnormally distributed variables, respectively. In all tests, p < 0.05 was considered statistically significant.

## 3. Results

There were 80 patients in Group 1, 80 patients in Group 2, and 85 subjects in the control group. As shown in Table 1, there were no differences between the groups in terms of age, sex, preoperative pterygium size and UV exposure (p > 0.05 for all comparisions).

The degree of inflammation was not different between the groups at both first month and 6 months after surgery (p = 0.208 and p = 0.428, respectively). However, in intergroup comparisons, a significant decrease in inflammation was observed between the first month and the sixth month in all three groups (p < 0.05) (Table 2).

There were no differences between the groups in terms of preoperative BCVA. A significant increase in postoperative BCVA was observed in all three groups compared to the preoperative period (p < 0.05) (Table 3).

Postoperative recurrence rates are shown in Table 4. The recurrence rate was determined by groups, sex, and preoperative pterygium size. There was no statistically significant difference between groups in terms of recurrence (p = 0.899).

Recurrence was seen in 3 females and 3 males in Group 1 (7.5%), in 2 females and 3 males in Group 2 (6.25%), and 4 females and 4 males in the control group (9.4%). There was no significant difference between the groups in terms of recurrence.

Patients under 40 years of age had the highest recurrence rate. This was not statistically significant (p>0.05). The number of recurrences decreased with advanced age. This situation was similar in all groups.

There were no statistically significant differences IOP levels between the groups. However, slight increases in IOP levels were observed in the groups between preoperative and postoperative sixth month follow-up measurements (p = 0.132 for Group 1, p = 0.350 for Group 2, and p = 0.142 for the control group respectively). The changes in IOP levels were not statistically significant when comparing across time and between groups (p = 0.818). It was observed that the increasing trend in IOP was consistent across all groups during the measurement periods.

Cases that developed complications were not included in this study. The inclusion and exclusion criteria are outlined above. Postoperatively, patients experienced symptoms such as burning, stinging, and dryness, and a persistent epithelial defect was observed in 8 patients. These symptoms regressed in all patients after 6 months of follow-up.

## 4. Discussion

In this study, we investigated the effects of 6 months of additional treatment with 0.05% CsA and 0.1% CsA on postoperative outcomes of patients with primary pterygium. We compared postoperative inflammation, BCVA, IOP, and recurrence rates between the groups. No statistically significant differences were found. These results indicate that different concentrations of CsA do not differ in their effectiveness in reducing postoperative pterygium recurrence.

Reviewing the literature, several studies suggest that the surgical technique we applied is the most successful pterygium surgical method with postoperative results [19]. The low recurrence rate and reduction in inflammation observed in the study groups confirmed the suitability of the conjunctival autograft technique. Moreover, previous research has shown promising results with the use of MMC in conjunction with conjunctival autografts for pterygium surgery [20,21]. For these reasons, these two methods were used in our study for optimal outcomes.

MMC inhibits cell proliferation and migrationand has applications beyond pterygium surgery, including ocular surface tumors, refractive surgery, glaucoma surgery, oculoplastic surgery, and strabismus surgery. While MMC is promising for these treatments, it can cause potential complications such as endothelial cell loss, corneal perforation, scleral melting, secondary glaucoma, iritis, and endophthalmitis. In our study, no side effects or complications were observed in any of the patients treated with MMC. This absence of complications may be attributed to the thorough washing of MMC, which was applied for three minutes during surgery and then extensively rinsed with at least 200 mL of serum irrigation.

The symptoms of pterygium are similar to those of dry eye disease and meibomian gland dysfunction (MGD), including dryness and irritation. Janson et al. found a significant relationship between pterygium size and dry eye symptoms. Pterygium has a negative correlation with tear film break-up time (TBUT) and Schirmer test result, but a positive correlation with corneal staining. It has been shown that pterygium can cause compression under the meibomian glands due to its contact with the palpebral conjunctiva. There are studies in the literature showing that ocular surface disease index score (OSDI), tear film break-up time test (TBUT), and Schirmer test results of the patients treated with cyclosporine A improved significantly [21]. Cyclosporine has also been beneficial for complaints of dryness, stinging, itching, and burning.

Considering the effects of CsA on postoperative results, our study had a small sample size compared to the calculated value. Despite the low number of 19 recurrence cases, it is believed that drawing definitive conclusions about recurrence rates may not be appropriate.

In the study conducted by Ozulken et al., it was observed that the use of cyclosporine A showed a significant decrease in the recurrance rate on 56 patients [14]. Similar to our study, patients were given cyclosporine A for 6 months after surgery. However, MMC was not used in their study, and a different surgical technique was employed. The overall recurrence rate in their study (14.2%) was higher than the recurrence rate observed in our study (7.7 %), which may be attributed to the effect of MMC adjunctive therapy and differences in the surgical techniques used. There was a lower recurrence rate in our study, but the decrease was not statistically significant.

Meneghim et al. demonstrated that the application of 0.05% CsA combined with 5-fluorouracil, administered 10 days before and 10 days after the rotational conjunctival flap technique, did not result in different recurrence rates between the control and case groups at 6 months. Their observed recurrence rate was higher than that in our study [22]. Although these results are consistent with the general results of our study, the use of 5-fluorouracil instead of MMC and the shorter duration of CsA administration in their study may explain the higher recurrence rate observed compared to ours. Dhar et al. reported that administering 0.05% CsA 4 weeks before and after surgery had no effect on the recurrence rate [23]. Other studies that found no effect of CsA also used different inclusion criteria, such as bilateral pterygium with >2 mm corneal invasion. On the other hand, Tok et al. reported a significantly lower recurrence rate with 0.05% CsA [24]. Similarly, Ahmed et al. showed low recurrence rates in the control groups compared to those treated with 0.05% CsA [25]. Both studies found statistically significant reductions in recurrence rates in the groups treated with 0.05% CsA. These differences may be attributed to variations in the dose of cyclosporine A used and the follow-up periods. Consequently, these variations between studies make it challenging to directly compare the results. A metaanalysis of seven studies involving 408 patients confirmed that the adjuvant use of CsA significantly reduces recurrence compared to the bare sclera technique alone. However, this metaanalysis also indicated no difference in the recurrence rate of pterygium between the conjunctival autograft technique with and without CsA and the rotational conjunctival flap technique [20]. This result is consistent with our study. Patients with complications were not included in this study, so complications were not evaluated. In the metaanalysis, the CsA group showed a significantly lower incidence of complications compared to the control group [20]. In our study, subgroup analysis showed that the recurrence rate was not related to the age, sex, or preoperative pterygium size of the patients. Han et al., who used the same surgical technique, also reported that sex and age did not significantly affect recurrence rates at one year [26]. Anguria et al. similarly found that age had no effect on recurrence [27], which is consistent with our results. However, they identified pterygium size as an important marker. Other studies suggest that a larger pterygium size or grade before surgery is an important indicator of postoperative recurrence [26]. The differences in pterygium recurrence reported in studies can be attributed to the different surgical techniques used. The low recurrence rate observed in our study could be due to the concurrent use of conjunctival autograft and the small sample size.

Our study has some limitations. Due to the retrospective nature of the study, the accuracy of the information obtained from the records can be questioned. The limited number of samples, 6-month follow-up, and sampling from a single center were among the factors limiting the generalizability of the results to the whole population.

The results of this study demonstrated that primary pterygium surgery using the conjunctival autograft technique combined with MMC is effective, resulting in a low recurrence rate. However, the main hypothesis—that the addition of two different concentrations of 0.05% CsA and 0.1% CsA administered for 6 months postsurgery would reduce the risk of recurrence—was not supported. The lack of observed effectiveness of cyclosporine A at different doses may be attributed to the already low recurrence rate achieved through the use of the appropriate surgical technique and adjuvant MMC. Future, more comprehensive studies may provide further insight into this issue.

## Figures and Tables

**Table 1:** Comparison of demographic and preoperative data of the groups.

	Group 1	Group 2	Control	p
	n = 80	n = 80	n = 85	
Age (years)	53.87±11.35	54.67±10.85	54.32±10.99	0.684
Sex (n) Male/female	42/38	44/36	45/40	0.717
Average UV exposure (hours)	3.0–3.5	4	3.5–4.0	0.224
Mean pterygium size (mm)	3.5 (3–4)	3.4 (3–4)	3.6 (3–4)	0.185

**Table 2:** Comparison of inflammation results between groups.

Inflammation	Group 1	Group 2	Control	p	
	n = 80	n = 80	n = 85	Intergroup	Intragroup
G0 (first month)	15	17	20	0.208	<0.05
G1 (first month)	31	29	25		
G2 (first month)	25	24	30		
G3 (first month)	9	10	10		
G0 (sixth month)	70	72	72	0.428	
G1 (sixth month)	6	4	6		
G2 (sixth month)	3	4	5		
G3 (sixth month)	1	0	2		

**Table 3:** Comparison of BCVA results between groups.

BCVA	Group 1	Group 2	Control	p	
	n = 80	n = 80	n = 85	Intergroup	Intragroup
Preoperative	0.27±0.19	0.26 ±0.18	0.27 ± 0.19	0.657	<0.05
Postoperative	0.26 ±0.19	0.24 ±0.18	0.26 ± 0.19	0.724	

BCVA (logMAR mean ± standard deviation).

**Table 4:** Comparison of recurrence rates between groups.

		Group 1	Group 2	Control	p	
		n = 80	n = 80	n = 85	Intergroup	Intragroup
Recurrence (n)		6	5	8	0.899	>0.05
According to preoperative pterygium size (n)	3 mm	2	1	3	0.999	
	3.5 mm	3	2	2		
	4.5 mm	1	2	3		
Sex (n)	Female	3	2	4	>0.05	
	Male	3	3	4		

**Table 5:** Recurrence distribution of groups by age.

Age (years)	Group 1	Group 2	Control	p
	n = 80	n = 80	n = 85	
under 40 (n)	3	4	3	>0.05
40–49 (n)	2	1	3	
50–59 (n)	1	0	1	
59 and above (n)	0	0	1	
